# Enzymatic Synthesis of Formate Ester through Immobilized Lipase and Its Reuse

**DOI:** 10.3390/polym12081802

**Published:** 2020-08-11

**Authors:** Yesol Baek, Jonghwa Lee, Jemin Son, Taek Lee, Abdus Sobhan, Jinyoung Lee, Sang-Mo Koo, Weon Ho Shin, Jong-Min Oh, Chulhwan Park

**Affiliations:** 1 Department of Chemical Engineering, Kwangwoon University, Seoul 01897, Korea; isss9511@naver.com (Y.B.); leejh9512@naver.com (J.L.); wkqh14@gmail.com (J.S.); 2 Department of Agricultural Engineering, South Dakota State University, Brookings, SD 57006, USA; abdus.sobhan@sdstate.edu; 3 Gyedang College of General Education, Sangmyung University, Cheonan 31066, Korea; dorgly@smu.ac.kr; 4 Department of Electronic Materials Engineering, Kwangwoon University, Seoul 01897, Korea; smkoo@kw.ac.kr (S.-M.K.); weonho@kw.ac.kr (W.H.S.); jmOH@kw.ac.kr (J.-M.O.)

**Keywords:** conversion, immobilized enzyme, lipase, octyl formate, reusability

## Abstract

Octyl formate is an important substance used in the perfume industry in products such as cosmetics, perfumes, and flavoring. Octyl formate is mostly produced by chemical catalysts. However, using enzymes as catalysts has gathered increasing interest due to their environment-friendly proprieties. In the present study, we aimed to identify the optimal conditions for the synthesis of octyl formate through immobilized enzyme-mediated esterification. We investigated the effects of enzymatic reaction parameters including the type of immobilized enzyme, enzyme concentration, molar ratio of reactants, reaction temperature, and type of solvent using the optimization method of one factor at a time (OFAT). The maximum conversion achieved was 96.51% with Novozym 435 (15 g/L), a 1:7 formic acid to octanol ratio, a reaction temperature of 40 °C, and with 1,2-dichloroethane as solvent. Moreover, we demonstrated that the Novozym 435 can be reused under the optimal conditions without affecting the octyl formate yield, which could help reduce the economic burden associated with enzymatic synthesis.

## 1. Introduction

Ester compounds have their own characteristic fragrances according to their type, which are similar to those found in nature, and are often used as artificial fragrance in various products such as cosmetics, perfumes, and spices [1]. Of these, octyl formate is a colorless liquid with a strong rose- or orange-like flavor, often used in the perfume industry. The World Health Organization has previously reported that using octyl formate as a flavoring agent is acceptable, approving its use in the food industry [2]. The food flavor market was evaluated at $13.31 billion in 2018 and is predicted to increase up to $19.72 billion by 2026, as the demand for various traditional and natural flavorings increases [3]. Therefore, the demand for octyl formate is also expected to continue to grow along with the upward predicted trend of the world flavor market.

Formate esters are mostly obtained by the reaction of formic acid with alcohol, to which it is common to add a chemical catalyst [4]. The cost of such chemical catalyst is low; however, the high temperature and pressure conditions that must be maintained throughout the reaction and purification process are associated with high costs. This is because of unnecessary byproduct generation, which pollutes the environment, while consuming a large amount of energy. Extraction of flavor esters from biological materials is often neglected due to their low yield. Nevertheless, production of flavor esters using chemical processes is not ecologically friendly and is toxic to humans. Past studies have used the biocatalytic process to produce flavors from natural sources instead of chemical methods. These processes, performed by ambient optical conditions, can selectively produce high purity flavors and employ optimized techniques that allow biocatalyst reusability to reduce the associated costs [4,5,6].

Biologically active enzymes are more costly than chemical catalysts, but they have the advantage of being environmentally friendly. They can lead to energy savings by reacting at lower temperature and pressure conditions while reducing residue formation [7,8]. Enzymatic-mediated production of flavor esters is an attractive process that allows to optimize several reaction factors such as concentration of reactants, reaction time, temperature, and activity of the immobilized enzyme [4,9,10]. In addition, when the immobilized enzyme is used, costs can be reduced by easy separation of the product and enzyme. Furthermore, awareness of environmental problems has increased, and environment-friendly processes have attracted attention because of regulations.

Lipase has been successfully used as a biocatalyst in the transesterification with organic solvents to produce flavor esters phenethyl formate [4], isoamyl acetate [11,12], isoamyl butyrate [13], geranyl acetate [14], citronellyl acetate [15], octyl acetate [16], and methyl butyrate [17]. Lipases can be isolated from animals, plants, and recombinant microorganisms, and are important enzymes in the food, surfactant, and pharmaceutical industries. Several researchers have explored lipase immobilization and mediated esterification under solvent-free processes for the synthesis of flavors (methyl butyrate, octyl acetate, among others) without the need of enzyme separation and solvent toxicity reduction [11,18,19].

A previous report described the use of immobilized *Candida antarctica* and porcine pancreatic lipases for the production of butyl acetate (pineapple flavor), ethyl valerate (green apple flavor), and isoamyl acetate (banana flavor) in n-hexane [20]. Herein, we explored the potential use of commercial immobilized lipases (Novozym 435, Lipozyme RM IM, and Lipozyme TL IM) for the enzymatic synthesis of octyl formate. In addition, lipase concentration, molar ratio of reactants, reaction temperature, and type of solvent were optimized, and the method of one factor at a time (OFAT) was applied to confirm the influence of each parameter. Reusability of immobilized lipase was also investigated to reduce the production costs of octyl formate.

## 2. Materials and Methods

### 2.1. Reagents

In this study, the immobilized lipases used were Novozym 435 (*Candida antarctica* lipase B immobilized on an acrylic resin), Lipozyme RM IM (*Rhizomucor miehei* lipase immobilized on an anionic resin), and Lipozyme TL IM (*Themomyces lanuginose* lipase immobilized on silica gel). All enzymes were purchased from Novozymes (Bagsvaerd, Denmark). The solvent reactants used were acetonitrile (99.5%), toluene (99.5%), and n-hexane (96%) purchased from Junsei (Tokyo, Japan), as well as acetone (99.5%), tetrahydrofuran (THF), 1,2-dichloroethane (99%), cyclohexane (99.5%), n-heptane (98%), and iso-octane (98%) purchased from Dae-jung (Gyunggido, Korea).

### 2.2. Methodology

**Figure 1:**
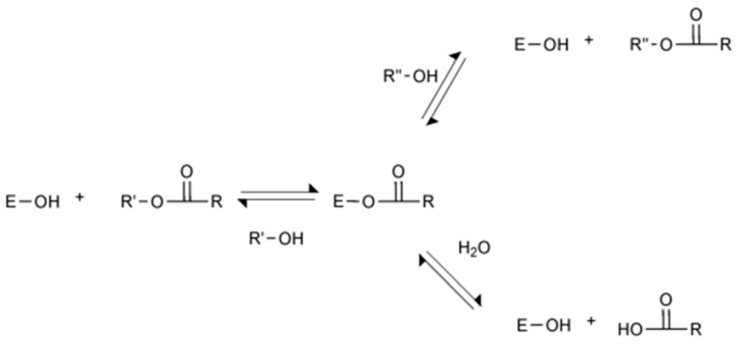
Lipase-catalyzed reactions.

Figure 1 shows the lipase-catalyzed reactions. The active site of lipase, serine hydroxyl group (E-OH), first reacts with the carboxylic acid or ester to form an acyl-enzyme complex. Afterwards, it reacts with nucleophiles such as alcohol and water [18]. Here, the type of immobilized enzyme, the enzyme concentration, the molar ratio of reactants, the reaction temperature, and the type of solvent were selected as the parameters affecting the octyl formate conversion. These variables were selected based on a previous literature review [11,12,13,14,15,16,17] and experiments were conducted by the OFAT method to find the optimal conditions. All experiments were performed twice.

### 2.3. Sample Preparation

Formic acid (100 mM) and octanol (100 mM) were each diluted in a selected solvent. The type of solvent was selected based on their hydrophobicity (referring to the log P): acetonitrile (−0.33), acetone (−0.16), THF (0.49), 1,2-dichloroethane (1.48), toluene (2.68), cyclohexane (3.40), n-hexane (3.76), n-heptane (4.27), and iso-octane (4.37). Diluted formic acid and octanol were mixed in a 1:1 ratio (*v*/*v*) and transferred to a serum bottle, with a final concentration of each substrate of 50 mM. Increasing concentrations of the different enzymes (5, 10, 15, 20, 25, and 30 g/L) were placed in the serum bottles, closed with a butyl injection stopper with cap headspace, and then punched. A shaking incubator (JEIO TECH.CO., LTD, Daejeon, Korea) was used for the catalytic reaction at a controlled temperature (20, 30, 40, or 50 °C), with an agitation of 150 rpm for 1 h, before sampling.

### 2.4. Enzyme Reuse

Each reaction per one cycle was carried out for 1 h under the defined optimal conditions—15 g/L of Novozym 435, 1:7 reactants molar ratio, reaction temperature of 40 °C, and 1,2-dichloroethane as solvent. After 1 cycle, the solution and the enzyme were separated using filter paper. The separated enzyme was washed with n-hexane and dried in a vacuum desiccator with silica gel for 1 h. It was then reused in the reaction up to a total of 10 cycles.

### 2.5. Sample Analysis

Analysis was performed by extracting 1 mL from each serum bottle and filtering the sample through a syringe filter (0.20 μm polytetrafluoroethylene (PTFE) membrane; Advantec Co., Tokyo, Japan) and storing it in a gas chromatography (GC) vial. The filtered samples were analyzed using the Agilent 7890A GC system (Agilent Technologies, Wilmington, DE, USA). The column used was an HP-INNOWax column (length 30 m, inner diameter 0.25 mm, film thickness 0.25 μm; Agilent Technologies, Wilmington, DE, USA) and the carrier gas was nitrogen. The oven was maintained at 80 °C for 1 min and then raised by 10 °C/minute up to 230 °C that was maintained for 3 min. Thereafter, the temperature was increased by 20 °C for 1 min and maintained at 250 °C for 1 min.

## 3. Results and Discussion

### 3.1. Effects of Experimental Variables on Enzymatic Synthesis of Octyl Formate

#### 3.1.1. Enzyme Selection

**Figure 2:**
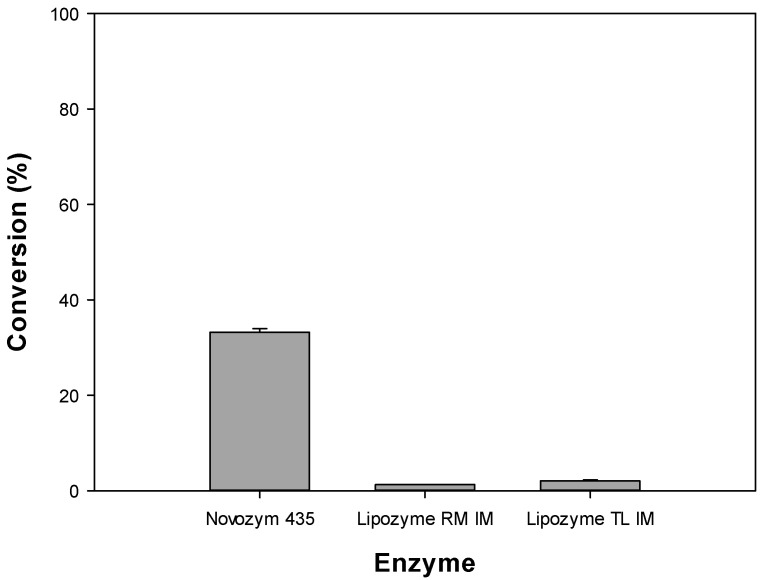
Conversion of octyl formate using commercial lipases.

The conversion of the immobilized enzyme, which was expected to have the greatest influence on the octyl formate conversion, was assessed using three commercially available immobilized enzymes—Novozym 435, Lipozyme RM IM, and Lipozyme TL IM. Initial conditions were set at 5 g/L, formic acid and octanol 1:1 ratio, 30 °C, with n-hexane as solvent. Novozym 435 showed a conversion of 33.23%, whereas Lipozyme RM IM and Lipozyme TL IM had almost equivalent low conversion of 1.28% and 2.09%, respectively (Figure 2), indicating that they were not suitable for converting octyl formate. Therefore, Novozym 435 was selected as the optimal enzyme for its good catalytic activity and was used in subsequent experiments (Table 1). Likewise, a previous report showed the Novozym 435, subjected to the derivatives of camelia oil, was significantly effective for the alcoholysis of camelia oil [21].

The catalyst used was deterministic for the rate of certain reactions due to the selectivity to the reactants. Among catalysts, enzymes have a high selectivity for reactants and this is referred to as the substrate specificity of the enzyme. Substrate specificity affects the rate of the reaction by reducing the potential energy barrier of the reactants. Table 1 shows that Novozym 435 is a non-selective lipase with high substrate specificity for esters and alcohols, whereas Lipozyme RM IM and Lipozyme TL IM, which are 1,3-position selective lipases, have substrate specificity to esters, making them more favorable for the esterification reaction. However, these commercial immobilized enzymes had distinctive activities and characteristics, and the Novozym 435 had significantly higher activity and broader substrate specificity, leading to higher conversion.

Novozym 435 has good catalytic activity on the transesterification reaction; however, it is important to control the additive amounts as this enzyme is expensive (2500 $/kg) [24], which could have a significant impact to the industrial process.

#### 3.1.2. Enzyme Concentration

**Figure 3:**
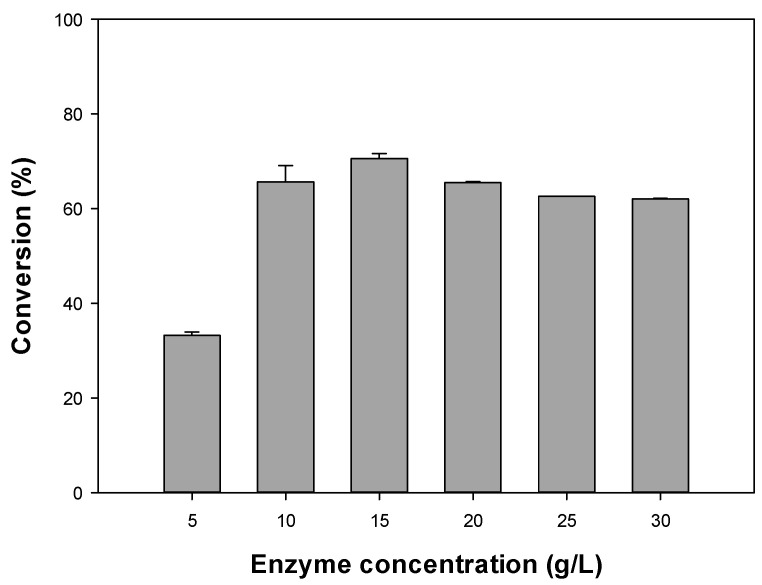
Effect of the selected lipase concentrations on the octyl formate conversion.

After identifying Novozym 435 as the optimal enzyme for the synthesis of octyl formate, we assessed the impact of different concentrations of the enzyme (Figure 3). At the initial 5 g/L enzyme concentration, the conversion was 33.23%, which increased to 65.64% with 10 g/L of enzyme, with 15 g/L of enzyme showing the highest conversion of 70.55%. The conversion decreased slightly at 20 g/L of enzyme to 65.49%, and furthermore at 25 and 30 g/L of enzyme. In accordance with our results, previous studies reported an increase in the conversion as the concentration of the lipase increased in the range of 10 to 30 mg/mL, followed by a reduction of the conversion as the concentration of the enzyme was further increased [25].

According to the Michaelis–Menten equation, the maximum constant reaction rate is determined by the initial enzyme concentration. As the enzyme concentration increases, the initial reaction rate increases rapidly, as well as the conversion. However, when the enzyme is in excess, a problem arises in the diffusion of the substrate [25,26], leading to a reduction in the conversion of octyl formate. This occurs due to the limited internal and external mass transfer associated with high enzyme loading [4,7,26,27]. The non-contributing behavior of excessive immobilized enzyme towards higher molar conversion, and associated reduced product yield, was also reported in the production of amyl isobutyrate [28] and citronellyl acetate [15]. Among all variables, the amount of immobilized enzyme had the greatest effect on attaining higher molar conversions. Therefore, the optimal enzyme concentration is key. For octyl formate production, the optimal concentration of Novozym 435 is 15 g/L, which we used in the following experiments.

#### 3.1.3. Molar Ratio of Reactants

**Figure 4:**
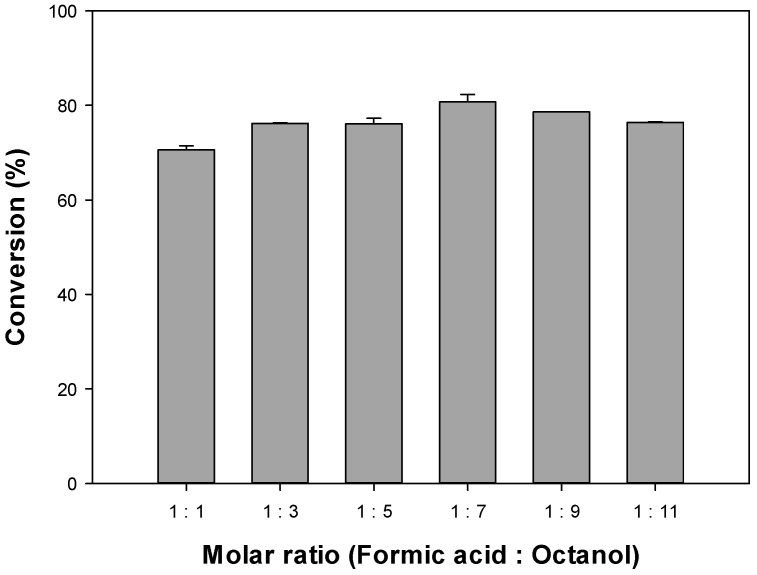
Effect of molar ratio of octyl formic acid and octanol on the octyl formate conversion.

Figure 4 depicts the impact of different molar ratios of formic acid to octanol on the conversion of octyl formate. The 1:1 molar ratio was associated with a conversion of 70.55%, while at molar ratios of 1:3, 1:5, and 1:7 were 76.17%, 76.07%, and 80.71%, respectively. The 1:9 and 1:11 molar ratios showed a slight decrease in the conversion with 78.60% and 76.40%, respectively. According to the lipase mechanism of action, formic acid first reacts with the lipase to produce an enzyme substrate complex, with which the alcohol reacts to generate an ester. When the alcohol is insufficient, only the enzyme substrate complex is formed but not the ester. In contrast, when alcohol is in excess, the alcohol reacts with the lipase first, and no ester is formed. Therefore, it is important to find the optimal molar ratio.

Herein, the highest conversion of octyl formate was found at the 1:7 formic acid to octanol ratio, and the subsequent experiments were conducted based on this result. Nevertheless, the conversion efficiency did not differ significantly as the amount of octanol increased. The esterification reaction is reversible and, when the presence of high water content, a hydrolysis reaction may instead occur, which can also be reversed [25]. Concentration of alcohols are a factor preventing activity of lipases and short-chain alcohols can irreversibly inactivate lipases, therefore the alcohol added is key to regulating the conversions [29].

#### 3.1.4. Effect of Temperature

**Figure 5:**
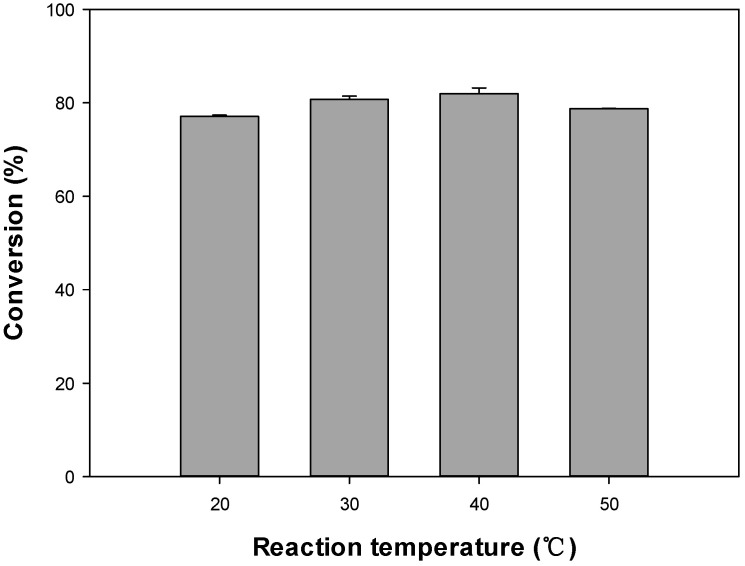
Effect of reaction temperature on the octyl formate conversion.

The impact of the reaction temperature on the lipase-catalyzed esterification of octyl formate was evaluated, with the temperature being tested at 20, 30, 40 and 50 °C in the view of the boiling point of the solvent and the economics of the process (Figure 5). The conversions were 77.10%, 80.71%, 81.96%, and 78.71% at 20, 30, 40, and 50 °C, respectively. The highest conversion (81.96%) was at 40 °C, therefore is the optimum reaction temperature. However, only a slight variation (≈4%) between lowest and highest conversion was observed; thus, the reaction temperatures tested had a low impact on the enzymatic activity. In a previous study, a conversion of 82% of octyl acetate was reported at 30 °C using Novozym 435 within 90 min [16]. To maintain high catalytic activity for a long time, mild reaction temperatures are recommended because Novozym 435 is a protein/enzyme that could be denatured at high temperatures [30].

As the temperature rises at high substrate concentrations, the solubility increases, and the conversion may also increase. Novozym 435 is a heat-resistant enzyme and has been reported to withstand 99.8 °C. However, at this high temperature it is rapidly deactivated; thus, reactions at lower temperatures are preferable, with the optimum temperature for Novozym 435 here being set between 30–60 °C.

#### 3.1.5. Variations in Solvent

The conversions dependence on solvent selection was also examined for octyl formate production. The solvent was selected using the log P value, which is the partition coefficient between octanol and water, and is often used to define the hydrophilicity of the solvent and the enzyme activity in the hydrophobic environment. In a highly hydrophilic solvent, the enzyme activity is impaired due to loss of the necessary fine water layer present in the enzyme, whereas in a hydrophobic solvent the water produced by the reaction cannot be removed from the lipase, which will prevent the reaction between lipase and the substrate [31,32,33,34]. Therefore, it is necessary to find a solvent having an appropriate log P.

**Figure 6:**
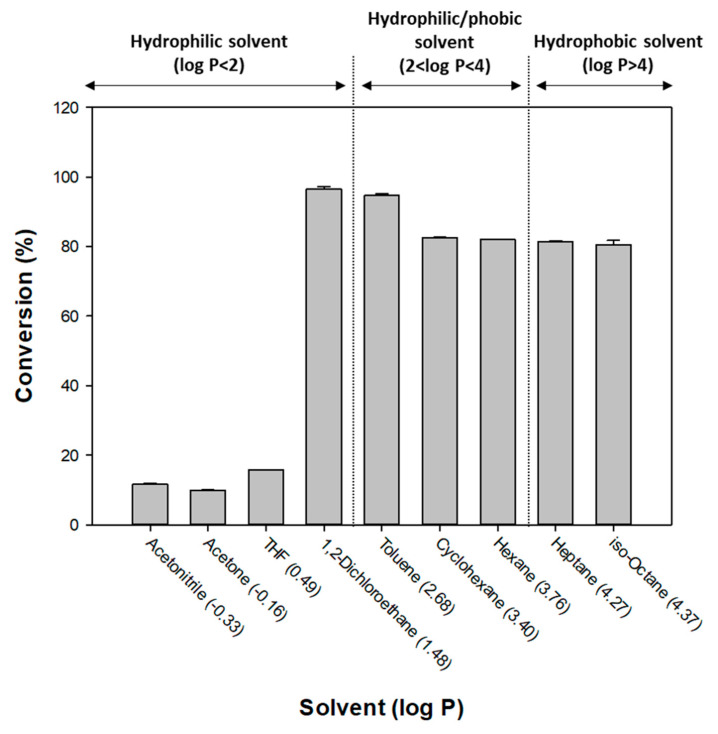
Conversion of octyl formate using various solvents.

Figure 6 shows the conversions for the different solvents tested. The hydrophilic solvents acetonitrile, acetone, THF, and 1,2-dichloroethane showed a conversion of 11.75%, 9.95%, 15.71%, and 96.51%, respectively, whereas toluene, cyclohexane, n-hexane, n-heptane, and iso-octane showed a conversion of 94.63%, 82.42%, 81.96%, 81.44%, and 80.39%, respectively. Therefore, 1,2-dichloroethane, which showed the highest conversion, was selected the as optimal solvent.

### 3.2. Reusability of Selected Lipase for Synthesis of Octyl Formate

Enzymes are very expensive, which will impact in the overall synthesis process costs. Therefore, an enzyme that can be recycled multiple times is more desirable for both industrial and laboratorial purposes [4,6]. To determine whether enzyme recycle could be applied to our reaction system, we performed the reusability experiment of the Novozym 435 under the defined optimal conditions.

**Figure 7:**
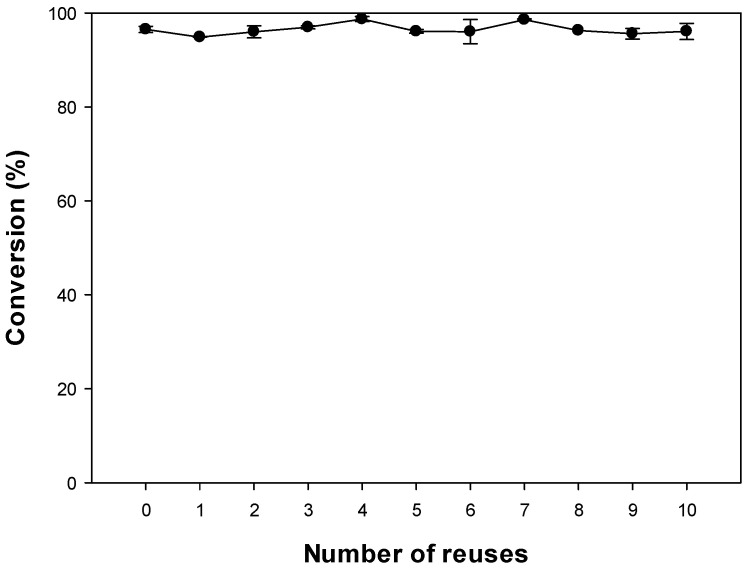
Reusability of selected lipase for high conversion of octyl formate.

A previous study reported that Novozym 435 expressed 30% higher activity than a new preparation, but the latter retained its higher degree activity during reuse [35]. In this study, the experiment was conducted under the optimal conditions detailed in Section 3.1.1, Section 3.1.2, Section 3.1.3, Section 3.1.4 and Section 3.1.5, and the conversion was maintained at ~94.86% for 10 reactions (Figure 7). Another study showed that the enzyme maintained its activity at ~70% of the initial activity after 14 reactions, even upon undergoing washing with n-hexane and drying [36], which is similar to the protocol we used. In addition, Novozym 435 assisted by toluene that was reused multiple times in esterification reactions showed to retain its activity with a conversion level maintained at a minimum value of 92% [4].

Enzymes have the disadvantage of being more expensive than chemical catalysts. To overcome this problem, we conducted and confirmed that enzyme reuse is possible for octyl formate conversion. However, considering the economic efficiency, our results can only provide a guide for esterification reactions where esters can be purified using Novozym 435.

## 4. Conclusions

The synthesis of octyl formate using enzymes can be conducted under milder conditions than conventional chemical methods while inhibiting pollutant byproducts, thus contributing to environmental preservation. Here, a series of reaction condition optimization procedures were performed to establish the optimal reaction conditions for octyl formate synthesis. Novozym 435 at a concentration of 15 g/L was added and the molar ratio of formic acid to octanol was set to 1:7, while 1,2-dichloroethane was used as the solvent. The reaction was conducted at the optimal temperature of 40 °C and 150 rpm in a shaking incubator. In addition, economic efficiency was considered, by reusing the immobilized enzyme in the reaction. Since these results were based on limited experiments, further investigation is still required for industrial application. Nevertheless, our results can pave the way for the environmentally friendly synthesis of octyl formate.

## Figures and Tables

**Table 1:** Characteristics of commercial lipases [22,23].

Immobilized Enzyme	Support	Activity	Optimal Temp. (°C)	Specific Substrate	Regioselectivity
Novozym 435	Lewatit vp oc 1600	10,000 PLU/g	30–60	Esters and alcohols	Nonspecific
Lipozyme RM IM	Duolite ES 562	275 IUN/g	30–50	Esters	1,3-specific
Lipozyme TL IM	Gel silicate	250 IUN/g	50–75	Esters	1,3-specific

LU—Lipase unit; PLU—Propyl laurate unit; IUN—Interesterification unit. 1 PLU is equal to 1 IUN.

## References

[1] Berger R.G., De Bont J.A.M., Eggink G., Da Fonseca M.M., Gehrke M., Gros J.-B., Van Keulen F., Krings U., Larroche C., Leak D.J., Swift K.A.D. (1999). Biotransformations in the flavour industry. Current Topics in Flavours and Fragrances: Towards a New Millennium of Discovery.

[2] Evaluations of the Joint FAO/WHO Expert Committee on Food Additives (JECFA). https://apps.who.int/food-additives-contaminants-jecfa-database/chemical.aspx?chemID=2565.

[3] Food Flavors Market to Reach USD 19.72 Billion by 2026|Reports and Data. https://www.reportsanddata.com/report-detail/food-flavors-market#utm_source=globenewswire&utm_medium=referral&utm_campaign=shuv10oct2019&utm_content=DP.

[4] Shin M., Seo J., Baek Y., Lee T., Jang M., Park C. (2020). Novel and efficient synthesis of phenethyl formate via enzymatic esterification of formic acid. Biomolecules.

[5] Gatfield I.L. (1995). Enzymatic and microbial generation of flavors. Perfum. Flavor..

[6] Garlapati V.K., Banerjee R. (2013). Solvent-free synthesis of flavour esters through immobilized lipase mediated transesterification. Enzyme Res..

[7] Cha H.-J., Park J.-B., Park S. (2019). Esterification of secondary alcohols and multi-hydroxyl compounds by *Candida antarctica* lipase B and subtilisin. Biotechnol. Bioprocess Eng..

[8] Won Y., Pagar A.D., Patil M.D., Dawson P.E., Yun H. (2019). Recent advances in enzyme engineering through incorporation of unnatural amino acids. Biotechnol. Bioprocess Eng..

[9] Janssen L.M.G., van Oosten R., Paul C.E., Arends I.W.C.E., Hollmann F. (2014). Lipase-catalyzed transesterification of ethyl formate to octyl formate. J. Mol. Catal. B Enzyme.

[10] Stergiou P.-Y., Foukis A., Filippou M., Koukouritaki M., Parapouli M., Theodorou L.G., Hatziloukas E., Afendra A., Pandey A., Papamichael E.M. (2013). Advances in lipase-catalyzed esterification reactions. Biotechnol. Adv..

[11] Ghamgui H., Karra-Chaâbouni M., Bezzine S., Miled N., Gargouri Y. (2006). Production of isoamyl acetate with immobilized *Staphylococcus simulans* lipase in a solvent-free system. Enzyme Microb. Technol..

[12] Torres S., Baigorí M.D., Swathy S.L., Pandey A., Castro G.R. (2009). Enzymatic synthesis of banana flavour (isoamyl acetate) by *Bacillus licheniformis* S-86 esterase. Food Res. Int..

[13] Hari Krishna S., Karanth N.G. (2001). Lipase-catalyzed synthesis of isoamyl butyrate. Biochim. Biophys. Acta.

[14] Claon P.A., Akoh C.C. (1994). Enzymatic synthesis of geranyl acetate in n-hexane with *Candida antarctica* lipases. J. Am. Oil Chem. Soc..

[15] Claon P.A., Akoh C.C. (1994). Effect of reaction parameters on SP435 lipase-catalyzed synthesis of citronellyl acetate in organic solvent. Enzyme Microb. Technol..

[16] Yadav G.D., Trivedi A.H. (2003). Kinetic modeling of immobilized-lipase catalyzed transesterification of n-octanol with vinyl acetate in non-aqueous media. Enzyme Microb. Technol..

[17] Kwon D.Y., Hong Y.-J., Yoon S.H. (2000). Enantiomeric synthesis of (S)-2-methylbutanoic acid methyl ester, apple flavor, using lipases in organic solvent. J. Agric. Food Chem..

[18] Singh P., Saxena D.K., Naik S.N. (2014). Synthesis of food flavors by enzymatic esterification process. Int. J. Sci. Res..

[19] Güvenç A., Kapucu N., Mehmetoğlu Ü. (2002). The production of isoamyl acetate using immobilized lipases in a solvent-free system. Process Biochem..

[20] Adlercreutz P. (2013). Immobilisation and application of lipases in organic media. Chem. Soc. Rev..

[21] Verdasco-Martín C.M., Villalba M., dos Santos J.C.S., Tobajas M., Fernandez-Lafuente R., Otero C. (2016). Effect of chemical modification of Novozym 435 on its performance in the alcoholysis of camelina oil. Biochem. Eng. J..

[22] SÁ A.G.A., Meneses A.C., Araujo P.H.H., Oliveira D. (2017). A review on enzymatic synthesis of aromatic esters used as flavor ingredients for food, cosmetics and pharmaceuticals industries. Trends Food Sci. Technol..

[23] Immobilized Lipase for Biocatalysis for Smart Chemical Synthesis, Novozymes. http://www.novozymes.com/en.

[24] Gu J., Xin Z., Meng X., Sun S., Qiao Q., Deng H. (2015). Studies on biodiesel production from DDGS-extracted corn oil at the catalysis of Novozym 435/super absorbent polymer. Fuel.

[25] Wang Y., Zhan D.-H., Chen N., Zhi G.-Y. (2015). Synthesis of benzyl cinnamate by enzymatic esterification of cinnamic acid. Bioresour. Technol..

[26] Yadav G.D., Devendran S. (2012). Lipase catalyzed synthesis of cinnamyl acetate via transesterification in non-aqueous medium. Process Biochem..

[27] Sun W.-J., Zhao H.-X., Cui F.-J., Li Y.-H., Yu S.-L., Zhou Q., Qian J.-Y., Dong Y. (2013). D-isoascorbyl palmitate: Lipase-catalyzed synthesis, structural characterization and process optimization using response surface methodology. Chem. Cent. J..

[28] Bezbradica D., Mijin D., Siler-Marinkovic S., Knezevic Z. (2006). The *Candida rugosa* lipase catalyzed synthesis of amyl isobutyrate in organic solvent and solvent-free system: A kinetic study. J. Mol. Catal. B Enzyme.

[29] Deng L., Xu X., Haraldsson G.G., Tan T., Wang F. (2005). Enzymatic production of alkyl esters through alcoholysis: A critical evaluation of lipases and alcohols. J. Am. Oil Chem. Soc..

[30] Liu S., Nie K., Zhang X., Wang M., Deng L., Ye X., Wang F., Tan T. (2014). Kinetic study on lipase-catalyzed biodiesel production from waste cooking oil. J. Mol. Catal. B Enzyme.

[31] Bovara R., Carrea G., Ottolina G., Riva S. (1993). Water activity does not influence the enantioselectivity of Lipase PS and lipoprotein lipase in organic solvents. Biotechnol. Lett..

[32] Lima V.M.G., Krieger N., Mitchell D.A., Fontana J.D. (2004). Activity and stability of a crude lipase from *Penicillium aurantiogriseum* in aqueous media and organic solvents. Biochem. Eng. J..

[33] Pan S., Liu X., Xie Y., Yi Y., Li C., Yan Y., Liu Y. (2010). Esterification activity and conformation studies of *Burkholderia cepacia* lipase in conventional organic solvents, ionic liquids and their co-solvent mixture media. Bioresour. Technol..

[34] Wang S., Meng X., Zhou H., Liu Y., Secundo F., Liu Y. (2016). Enzyme stability and activity in non-aqueous reaction systems: A mini review. Catalyst.

[35] Graebin N.G., Martins A.B., Lorenzoni A.S.G., Garcia-Galan C., Fernandez-Lafuente R., Ayub M.A.Z., Rodrigues R.C. (2012). Immobilization of lipase B from *Candida antarctica* on porous styrene-divinylbenzene beads improves butyl acetate synthesis. Biotechnol. Progress.

[36] Martins A.B., Schein M.F., Friedrich J.L.R., Fernandez-Lafuente R., Ayub M.A.Z., Rodrigues R.C. (2013). Ultrasound-assisted butyl acetate synthesis catalyzed by Novozym 435: Enhanced activity and operational stability. Ultrason. Sonochem..

